# Older adults’ experiences of hospital-to-home transitions in rural Sweden: a qualitative study

**DOI:** 10.1186/s12877-025-06780-1

**Published:** 2025-12-02

**Authors:** Idun Byberg, Ulla Näppä, Marie Häggström

**Affiliations:** 1 Department of Health Sciences, Mid Sweden University, Östersund, S-831 25 Sweden; 2 Department of Health Sciences, Mid Sweden University, Sundsvall, S-851 70 Sweden

**Keywords:** Care transition, Coordinated care, Experiences, Hospital to home transition, Older adults, Patient discharge, Patient transfer, Rural areas, Rural nursing, Qualitative research

## Abstract

**Background:**

The transition from hospital to home represents a pivotal and potentially high-risk phase for older adults, especially within rural contexts where geographical remoteness, limited resources, and decentralized healthcare infrastructure amplify vulnerabilities. Existing literature offers limited insight into the experiences of rural older adults during this transitional process. Therefore, this study aimed to explore older adults’ experiences of transitioning from hospital to home in rural settings and to describe how they felt during the process.

**Methods:**

This qualitative study used open, unstructured interviews to explore older adults’ experiences of hospital-to-home transitions in a rural region of northern Sweden. Nine older adults (aged 69–85) who had recently been discharged from somatic inpatient wards of a county hospital participated. In one interview, the spouse of one of them also participated. Data were analyzed using a six-phase Reflexive Thematic Analysis.

**Results:**

Older adults’ hospital-to-home transitions were experienced as twofold, involving both a physical transition from hospital to home and an inner transition of self-image. Their experiences related to three interconnected dimensions: organizational, psychological, and social. The themes identified were “Knowing one’s way through the healthcare system,” “Understanding and managing one’s thoughts and emotions,” and “Feeling socially connected.”

**Conclusions:**

Care transitions for older adults in a rural context are complex and highly personal; therefore, healthcare professionals must thoroughly assess each individual’s specific circumstances, including their psychological resources and social networks, during care planning.

**Supplementary Information:**

The online version contains supplementary material available at 10.1186/s12877-025-06780-1.

## Introduction

Care transitions from hospital to home [1, 2], especially for older adults^1^[3], are risky processes where individuals with complex conditions are most at risk [4, 5]. Understanding the transition experience, including losses, gains, and transformations, is crucial for nursing [1, 2]. Actively involving older adults during care transitions presents significant challenges [6]. The organization of care transitions often stresses and confuses older adults, putting them in vulnerable positions [7, 8]. In Sweden, older adults report feeling excluded from discharge planning and care coordination, despite being central to the process [9]. This study focuses on older adults’ experiences of care transitions from hospital to home in a rural Swedish context.

## Background

Frail older adults often find the transition from hospital to home more unsafe and troublesome than their less frail counterparts [10]. Both older adults and their relatives, whom the older adults often rely on during discharge processes [11], may perceive care transitions from hospital to home as filled with exhaustion, frustration, and uncertainty [12]. Their level of satisfaction is linked to care quality [13]; however, the degree of their participation before discharge is not always satisfactory [14, 15]. Older adults have complex care paths after hospital discharge [16], and in Scandinavia, the main path is back to home with or without home healthcare [17].

The Swedish healthcare system is primarily tax-funded, and care is generally provided free at the point of delivery, with minor nationally regulated fees [18]. The system is characterized by decentralization, structured across three main political levels, (1) the national government, (2) 21 regional councils responsible for hospital and specialist care, and (3) 290 municipalities managing home healthcare and social services [19, 20]. This division necessitates close collaboration across political levels and organizational boarders, particularly during care transitions from hospital to home, which can be complex especially in rural areas [21]. Ingvarsson’s [9] study in urban southern Sweden emphasizes systemic communication gaps and poor documentation of older adults’ preferences. These issues are likely amplified in rural areas due to resource constraints, geographic isolation, and the decentralized governance described by Carson et al. [22], leading to fragmented accountability among healthcare providers.

The care environment is central to healthcare, encompassing the setting and requirements for practice [23]. Environmental factors such as resources, accessibility of services, and healthcare policies significantly influence the experience of care transitions [24]. These factors often vary between urban and rural contexts [25–27], and challenges associated with rural hospital-to-home transitions are found worldwide, including in Sweden. For instance, transportation barriers, a challenge heightened in rural, sparsely populated areas [28, 29], decrease the likelihood of follow-up completion [30]. In Sweden’s rural context, geography worsens issues related to distance, service availability, and staffing shortages, while decentralized governance divides responsibilities among regional hospitals and municipal care providers (20- the Swedish law on healthcare responsibilities, 22). Transportation barriers [28, 30] and resource constraints [24] in rural areas are particularly critical in Sweden, which has the lowest hospital bed ratio in the European Union [31]. The decentralized system intensifies pressure on care transitions, especially in rural regions where long distances, sparse populations, and limited resources complicate coordination [21, 22].

A scoping review of rural hospital-to-home transitions [32] included only two Swedish studies: one on first-time mothers [33] and another combining urban and rural older adults [34]. Most studies exploring older adults’ perspectives on care transitions from hospital to home either lack rural specificity or fail to contextualize findings within the challenges facing rural areas [32]. This gap is critical, as older adults’ satisfaction, a key quality metric [35], requires more profound insights into how rural structures and processes influence outcomes. For instance, Ingvarsson’s [9] findings on urban patient disempowerment suggest that rural older adults may encounter compounded vulnerabilities due to systemic fragmentation and isolation.

Meleis [1, 36] Transition Theory provides a comprehensive framework for understanding the processes that older adults undergo during changes in health status, such as transitions from hospital to home. Transitions are complex and multidimensional processes encompassing situational changes like discharge, developmental health-illness, and organizational transitions, often overlapping and interacting [37]. These transitions are marked by uncertainty, vulnerability, and the need for adaptation, putting older adults at risk for adverse outcomes if not supported. Within this framework, healthcare professionals are crucial; they identify facilitators and barriers while providing interventions to promote healthy transitions and enhance well-being [36]. This involves collaborating with older adults, ensuring continuity, and supporting the development of coping strategies and self-management skills. Transitional care aims to facilitate healthy transition processes [38] by ensuring safe, effective transfers and addressing the psychological, social, and educational needs that arise [39].

Sweden’s unique combination of low hospital bed availability, decentralized healthcare governance, and rural geography creates a perfect storm of challenges for older adults during care transitions, in which their experiences remain critically underexplored [32]. In Sweden’s rural context, geography worsens issues related to distance, service availability, and staffing shortages, while decentralized governance divides responsibilities among regional hospitals and municipal care providers [20, 22]. Similar challenges are faced by rural health systems worldwide, highlighting the broader relevance of this study and its findings beyond Sweden.

This study aimed to explore older adults’ experiences of transitioning from hospital to home in rural settings and to describe how they felt during the process. The research questions were: What do older adults in rural Sweden think of the organization of care transitions? What feelings arise during this process, and how do they manage them?

## Methods

This explorative, qualitative interview-study was grounded in the ontological assumption that the world is socially constructed, subjectively perceived, and multifaceted. Epistemologically, this implies that there is no single truth. Meanings are seen as co-created by the researcher and the participants, and knowledge is contextually situated to time and place [40]. Given the study’s aim, we interviewed older adults to gain insight into their unique experiences and perspectives on care transitions in a rural region of Sweden. We analyzed the data using the 6-step Reflexive Thematic Analysis proposed by Braun and Clarke [40, 41], a flexible qualitative method aimed at identifying and interpreting meaningful themes across the data set. Reflexive Thematic Analysis aligns with a social constructionist epistemology, acknowledging the researchers’ active role in co-constructing meanings through an iterative, reflexive analytic process. Study reporting was supported by the Reflexive Thematic Analysis Reporting Guidelines (RTARG), as recommended by Braun and Clarke [42].

The research team consists of registered nurses with different specialist backgrounds (district nurse, oncology nurse, intensive care nurse/acute care nurse). Our previous research on transitional care quality and professional collaboration in the same region has contributed to a shared pre-understanding of the context influencing older adults’ transitions. During this study, including design, data collection, and analysis, no real disagreements arose among us, which is likely due to our multiple perspectives and experiences contributing to a nuanced and reliable interpretation of the data. Reflexive memos written by I.B.^2^ were continuously discussed within the research team, including considerations related to the potential influence of holding dual roles as both researcher and district nurse. These discussions contributed to maintaining ethical awareness and analytic reflexivity throughout the research process, particularly in interview situations where professional boundaries and participant expectations required careful navigation. Examples of reflexive memos on data analysis and data collection, and how mutual discussions on preunderstanding formed the research process, are shown in Appendix 1.

### Setting and recruiting

This study was conducted in a northern region of Sweden where most municipalities are categorized as sparsely populated rural municipalities [43]. The region ranks among the top six least densely populated areas in the European Union [44]. Healthcare services are primarily provided by a central county hospital, which offers specialist care at the county level. For advanced specialized treatments, such as specific surgical procedures, older adults are referred to a university hospital about 400 km away that provides advanced specialized treatment for more regions. This university hospital functions as a tertiary care center, serving a larger geographical area and offering highly specialized services unavailable at the county hospital.

Inclusion criteria for the study were adults aged 65 years or older residing in the rural region who had recently transitioned or were about to transition from the county hospital to their homes. All older adults were required to have sufficient cognitive ability to provide informed consent and reflect on their experiences during their care transition. Formal cognitive assessment was not performed; eligibility was determined through the clinical judgment of healthcare professionals and the research team regarding the older adults’ comprehension of study information and consent procedures. Before enrollment, the older adults received both oral and written information and could clarify any questions, supporting the integrity of informed consent. During interviews, participants additionally evidenced the capacity to reflect on and articulate their experiences.

Older adults were recruited through healthcare professionals at somatic care clinics and home care services, as well as through community outreach. Due to initially low response rates, primarily attributed to the limited number of eligible individuals, additional recruitment efforts involved collaboration with pensioners’ associations, the distribution of study advertisements at healthcare centers, and posts on social media. Of those who expressed interest, one individual was excluded for being under 65, and two later declined participation due to feeling overwhelmed by their health conditions. The majority (*n* = 5) of the nine participating older adults reported having found the study advertisement via social media posts, while the remaining (*n* = 4) received information through healthcare professionals.

### Participants and data collection

Nine older adults who had recently been cared for in somatic inpatient wards at the county hospital consented to participate in open, unstructured interviews. The sample included three men and six women, aged 69 to 85 years (median age 75). The distance from older adults’ homes to the county hospital ranged up to 230 km, with an additional 26 km to the nearest primary care center. All had been admitted to somatic inpatient wards for medical, orthopedic, or surgical reasons. Two older adults’ discharges were preceded by care planning for home health services, and one was discharged to a short-term care unit before returning home. All received follow-up care through primary care or outpatient clinics after discharge. Regarding living arrangements, three older adults lived alone, and six lived with a spouse.

According to the ‘Rules of thumb’ (Sharma et al., 2024), we utilized the information power model presented by Malterud et al. [45] to estimate the required sample size, as recommended by Braun and Clarke [40]. This study was characterized by a well-defined, contextually grounded aim and specific participant attributes, underpinned by a theoretical framework drawing on Meleis’ Transition Theory [1] as well as relevant prior research. The data collection featured intensive, in-depth dialogue and reflexive analysis focusing on older adults’ narratives. The research group regularly reviewed the data to confirm that the study objectives were sufficiently addressed, and the emerging themes provided rich and relevant insights, ensuring adequate information power. According to Braun and Clarke [40], information power refers to the adequacy of the data in providing meaningful answers to the research questions, emphasizing depth and relevance over sample size. By revisiting the assessment stepwise during recruitment, reflexive memo writing, and critical discussions, we concluded that the sample provided sufficient information power.

Data collection occurred between September 2024 and March 2025. Interview methods were customized to older adults’ preferences (face-to-face or phone, individually or accompanied by a close relative) to ensure their comfort during the interview process. Eight older adults were interviewed individually, while one chose to be interviewed in pairs with their 91-year-old spouse, who was also involved as a close relative in the care transition. They had been married for 65 years and completed each other’s sentences, so some of what the spouse said was also included in subsequent analysis.

The timing of the interview regarding hospital discharge affected the number of interviews conducted. Three older adults underwent two to three interviews, either before or within a week of discharge, as they were recruited early in the transition process by healthcare personnel during the hospital stay and agreed to participate during or shortly after. The focus in the follow-up interviews was neither on temporality, time, or change, such as with a qualitative longitudinal research design [46], but rather on capturing their post-discharge experiences. These interviews were conducted both in person and by phone. The other six older adults were interviewed once, following an initial phone contact to schedule the interview and gather brief information about their hospitalization, which generated field notes providing contextual understanding for subsequent interviews. Among these six, three chose in-person interviews at a location of their preference, while three opted for phone interviews. Altogether, apart from initial phone contact for interview scheduling, 13 recorded interviews were conducted, of which six were face-to-face and seven were by phone.

The interviews were audio-recorded for later transcription and analysis. Audio recordings lasted between 41 and 144 min per older adult (median = 86 min). Each interview began with the older adults receiving verbal information about the study, including its purpose, voluntary nature, and data management procedures, which included confidentiality and storage. I.B. utilized a topic guide, created by all authors, serving as a checklist to ensure that relevant aspects were covered, as noted by Patton [47]. This flexible support tool was developed based not only on the study´s aim, research questions, and methodological literature [47, 48], but also on literature within the research field to ensure relevance and contextual alignment with existing knowledge. The topics to cover were the following:

- Health, previous healthcare experiences, and hospital stay.
- Involvement/participation regarding discharge/planning.
- Need for help after discharge.
- Practical information and contacts before and after discharge.
- Psychosocial and emotional management before, during, and after discharge.

Besides demographic inquiries, the topic guide entailed questions such as: “Can you describe your hospital stay; what led to your hospitalization?“, “At what point during your care did someone begin discussing discharge with you?“, and “How does/did it feel to return home?” Specific questions were not needed in every interview, as the older adults often shared their experiences spontaneously. In those situations, the conversation featured prompts like “How did you experience that?“, “What happened then?” and similar inquiries to gain a deeper understanding of the older adults’ narratives. At the end of each interview, I.B. summarized what seemed to be functioning well and what was less effective during the care transition, stating, “So you thought that … was functioning well, but … was functioning less well, is that correctly understood?” This approach ensured that stories were accurately interpreted and the key elements for an optimal care transition were captured and understood.

### Analysis

Data analysis employed the six-phase Reflexive Thematic Analysis process outlined by Braun and Clarke (2006, 2022), emphasizing reflexivity and the active, iterative development of themes as patterns of shared meaning underpinned by a central organizing concept, rather than by frequency (Braun & Clarke, 2022). This method aligns with our social constructionist epistemology (see Methods) and encompasses both semantic and latent meanings in the older adults’ narratives [40, 49]. The 6-step analysis is illustrated in Table 1, showing the iterative, reflexive process.

**Table 1:** Reflexive thematic analysis process of the study

Phases named in literature (40, 41)	Phases carried out in this study, guided by cited literature
PHASE 1 Familiarizing yourself with your data	I.B. began familiarization during initial contact and interview scheduling, as reasons for care transitions emerged early. Familiarization included conducting and transcribing interviews, writing reflexive memos, and repeatedly reading transcripts. Regular meetings with M.H^a^ and U.N^b^ ensured ongoing discussion of data and recruitment.
PHASE 2 Generating initial codes	Transcripts were imported to NVivo (v13, Lumivero, 2020) and coded by I.B. in Swedish, semantically and latently, based on the study aim. For example, from “It’s about 220–230 kilometers…”, the semantic code “Viewing 200 kilometers as an affordable distance” and latent codes like “resilience shaped by rural living” were constructed. See Appendix 1, Table A1 for more examples of interview text->semantic/latent code, later to be sorted into the same theme, supported by reflexive memos. An active decision was made to code using the first person (“I”) rather than the third person (“they”), in order to analytically remain as close as possible to the older adults’ own perspectives and to honor the subjectivity and authenticity of their narratives. Codes and data were shared with M.H. and U.N. to ensure soundness.
PHASE 3 Searching for themes	I.B. used PowerPoint (v2504, Microsoft, 2025) to map and connect codes into initial themes, defined as patterns of shared meaning with a central organizing concept (see Appendix 1, Figure A1). For example, the emerging theme “Understanding and managing one’s thoughts and emotions” included codes related to coping with uncertainty and illness, and to adapting to life after the inpatient care period. Analytical memos (examples shown in Appendix 1, Table A1) supported theme construction. Initial thematic maps were shared with M.H. and U.N. for feedback.
PHASE 4 Reviewing themes	Initially mapped codes were sorted into theme files in NVivo, with ongoing review and adjustment, supported by memos. The team regularly discussed pre-understandings and prior experiences to avoid over-interpretation.
PHASE 5 Defining and naming themes	This phase overlapped with others, as themes were refined and named, and some codes were excluded if they did not contribute to the theme’s meaning.
PHASE 6 Producing the report	The report was written using Word, PowerPoint, and NVivo, with repeated reference to all analytical material. Throughout, texts were shared with M.H. and U.N. for critical feedback. The team engaged in reflexive dialogue to challenge assumptions and ensure themes were grounded in the data and study aim. All excerpts were pseudonymized for ethical reasons (“OA” for “Older Adult” plus participant number).

^a^Marie Häggström

^b^Ulla Näppä

One older adult chose to be interviewed along with their spouse. Analytically, the spouse’s contributions were treated as contextual input that enriched the older adult’s account, rather than as an independent data source. For example, occasions when the spouse gave additional detail or reinforced elements of the narrative were integrated into the analysis, but always in relation to the older adult’s central experience (see Appendix 1, Table A1 for illustration). Potential power dynamics were considered, and care was taken to ensure that both voices were present in the narrative, with the older adult retaining primary agency over the story. Interview facilitation encouraged open dialogue and mutual engagement, with attention to the couple’s interaction style, which proved supportive rather than dominating or silencing.

Interviews and initial coding were conducted in Swedish. Quotations selected for inclusion in the results section were translated into English during the final stages of analysis and manuscript preparation, ensuring that translation was informed by both the analytic context and the original narrative meaning. All translated excerpts were carefully reviewed for accuracy and equivalence of meaning by the whole research team. Given that the study region has distinctive dialectal expressions uncommon in standard Swedish, two researchers (I.B. and U.N.) with local roots and linguistic familiarity contributed to clarifying and validating any region-specific terms during translation. This collaborative approach enhanced the trustworthiness of the translations and ensured a nuanced understanding of local and generational language use.

In qualitative research, an appropriate number of quotations is essential to illustrate and support themes, but an excess of citations can dilute the analytic depth, while too few quotations may reduce transparency and credibility. We have aimed to strike this balance following established methodological guidance (e.g., Braun & Clarke, 2006; Nowell et al., 2017), ensuring core contributions from participants are reflected while maintaining a focused and coherent narrative.

### Ethical considerations

This study’s performance was based on the recommendations in the Declaration of Helsinki [50] and by the Northern Nurses’ Federation [51]. Before providing their verbal and written consent, the older adults received verbal and written information about the study, including their right to withdraw their participation at any time without giving a reason. Regarding the participant who chose to be interviewed along with their spouse, separate consents were provided. As recruitment partly went through healthcare personnel, it was emphasized that participation was separate from the older adults’ care, and they were assured that choosing to participate or not would not affect their current or future treatment or follow-up within healthcare services. Data management for this study followed the Mid Sweden University’s established data management plan (Dnr MIUN 2024/1721) in accordance with the university’s guidelines and applicable GDPR regulations. Audio recordings of interviews and their transcripts were securely stored and archived digitally within the university’s regulated systems. Valuing their comfort and integrity, the older adults were given the option to choose the time and place for the interview, and their names have been pseudonymized (OA^3^ 1–9).

## Results

The older adults viewed care transitions as life events rather than just moments of transfer from hospital to home. This process included the onset of illness and the journey toward recovery. It involved moving from a well-being former self to an ill-being self, either rehabilitating back to a well-being state or accepting the new self, shaped by the past. The essential aspects of this, which influenced the experienced quality of the care transition, are presented through three interconnected themes. Their dimensions are organizational, psychological, and social, explaining that the transition was twofold: one being the physical transition from hospital to home, and the other the inner transition of self-image. The three themes explaining the older adults’ experiences were “Knowing one’s way through the healthcare system”, “Understanding and managing one’s thoughts and emotions”, and “Feeling socially connected”.

**Fig. 1:**
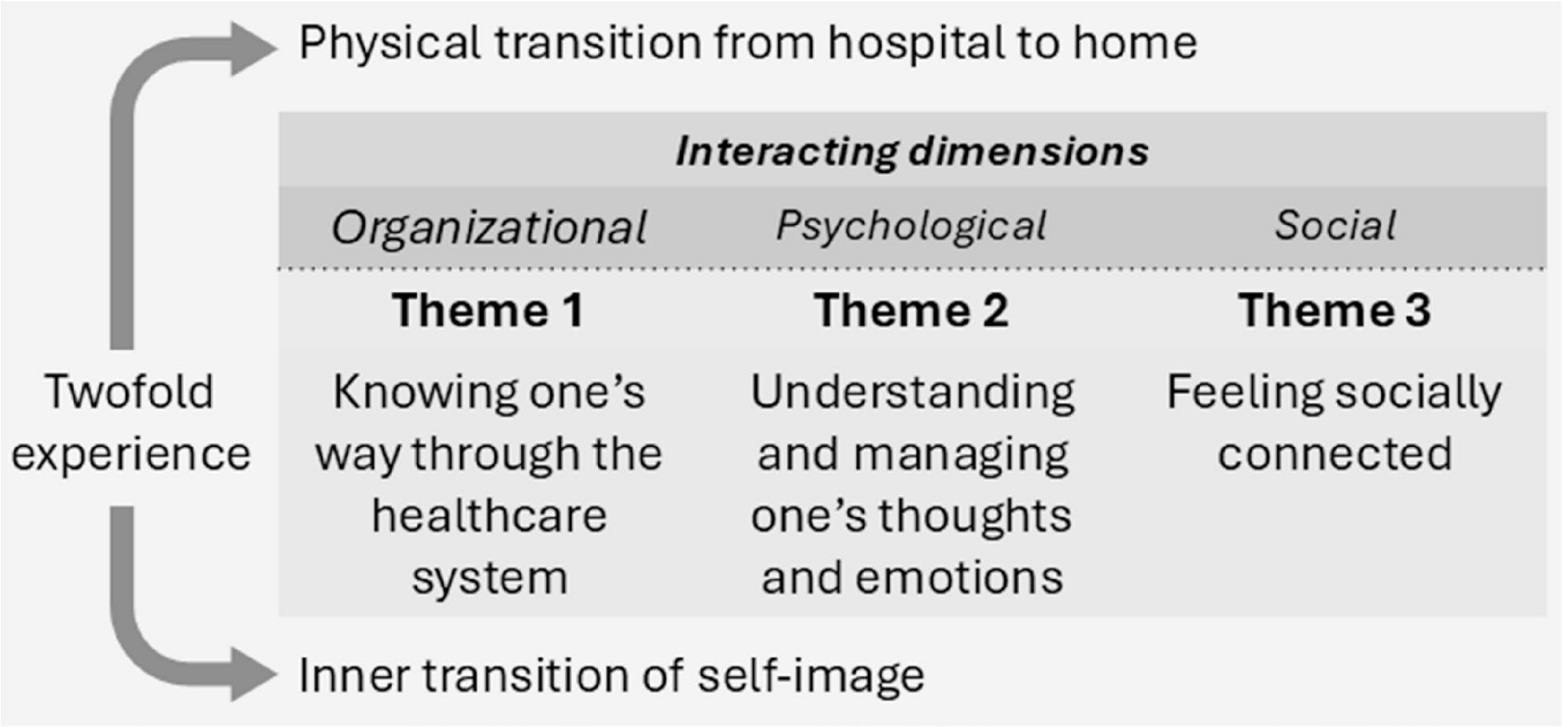
Dimensions and themes

The three thematic dimensions are deeply interconnected in shaping the care transition experiences among older adults. A strong social network (theme 3) facilitates navigation within a fragmented healthcare system (theme 1) and provides crucial emotional support for managing vulnerability and motivation (theme 2). Similarly, a positive psychological outlook and acceptance (theme 2) assist with engaging in healthcare structures (theme 1) and sustaining social relationships (theme 3). Understanding system navigation (theme 1) alleviates psychological challenges (theme 2) and prepares individuals for upcoming transitions. Recognizing system limitations (theme 1) underscores the importance of social networks (theme 3) to bridge gaps, such as arranging transportation when needed. Thus, themes 1–3 do not operate in isolation but interact dynamically to co-shape the quality and trajectory of each transition in this rural Swedish context. The dimensions and themes are shown in Fig. 1.

### Theme 1, Knowing one’s way through the healthcare system

This theme emphasizes the importance and critical need for older adults to understand and navigate the healthcare system. This includes knowing what to expect, how to access care, and how to participate in their care. The older adults highlighted the essential role of system navigation in addressing coordination issues, geographic barriers, and information gaps. Previous experiences—whether from prior healthcare interactions, those of close relatives, or having worked in healthcare—made it easier to navigate the system and know what questions to ask. The availability and continuity of healthcare personnel were crucial, especially in rural areas where long distances and limited resources often complicated access to care. The sparsely populated environment of a small community allowed individuals to personally know the healthcare personnel, fostering established relationships with them.

*“I’m thinking of two doctors who have been here [at the Primary Care centre]*,* they were classmates with our oldest daughter. So as children*,* they were at our place […] I think of her who is a doctor here now. Her parents were good friends with us. So her mother was our babysitter.”* (OA2).

The organization of care, including communication and coordination among different providers, played an important role in how safe and supported the older adults felt during care transitions. Several described how unclear information or conflicting messages from various professionals led to confusion and a feeling of being “bounced around” (OA9) in the system. The older adults said they had to manage their care even when they lacked energy or knowledge, which caused confusion and suffering. For example, physicians and nurses sometimes gave conflicting advice about whether discharge was appropriate, leaving the older adult responsible. Even when actively involved in this decision, it could, for example, result in an older adult returning home constipated due to their lack of knowledge about their condition.

*“When I was about to go home*,* it was tough. The doctor said*,* ‘You can go home if you want*,*’ and of course I did. But the nurse said*,* ‘No*,* I don’t think she should go home yet.’ In the end*,* I decided to leave. But I hadn’t managed to*,* well*,* I couldn’t have a bowel movement. I was so constipated*,* it’s hard to describe. The nurse probably saw that I wasn’t ready*,* but I went home anyway.”*(OA5).

The older adults highlighted the importance of being involved in planning and receiving information tailored to their needs and abilities. This was especially important for individuals without prior experience in the healthcare system to prevent feelings of confusion. The way information was delivered also mattered; a negative interaction could lead to mistrust about future care, while a friendly approach boosted a sense of security. Additionally, older adults showed different levels of interest in actively participating in decision-making. Some wanted to influence decisions, such as declining proposed care or co-deciding the timing of an upcoming procedure, while others preferred to stay well-informed and involved without taking on decision-making, clinical tasks, or managing communication between the hospital and primary care.

*“Sometimes I had to explain what they [primary care] should do. But I don’t know. I can’t tell you how to do the flushing. I can say that it needs to be done*,* but not how. That felt a bit much. You really shouldn’t place that kind of responsibility on the patient…”* (OA1).

Some older adults, especially those with professional healthcare experience, found it easier to navigate the system and advocate for their needs. Others described fighting for the care they believed they were entitled to, which could be exhausting when already weakened by illness. Experiences of not being listened to, taken seriously, or not receiving help for conditions that later proved serious left deep marks of anger and disappointment, leading to mistrust of the healthcare system.

*“I had sought help several times and was ignored.”* (OA4).

*“I am very critical of the healthcare system in general.”*(OA3).

The patient transport system was a key part of the care transition experience. Several older adults expressed frustrations with the lack of coordination between healthcare professionals and the department responsible for patient transport, which sometimes resulted in physically and emotionally exhausting journeys or even denied transport despite medical restrictions. These challenges were especially common in rural areas, where long distances and limited transport options made accessing care more difficult.

*“When I was going home*,* I wasn’t allowed to lift or do anything—not even my bag. So I called patient transport services. Nevertheless*,* do you think I got any help? No. I have no faith in them*,* it’s the worst imaginable. I will never contact them again.”*(OA9).

*“If you look at it from a healthcare perspective*,* [county name] is a very large region. Not everyone can just hop on and off the patient transport bus.”* (OA4).

Older adults valued different aspects of care in various ways during the transition process. For some, access to information and a sense of control were decisive for viewing the care transition as successful, while others found continuity and regular contact with familiar healthcare staff most reassuring. Access to one’s electronic health record could provide reassurance for some, but also lead to frustration when mistakes or shortcomings were revealed and not addressed.

*“… I think they made a mistake. They wrote a lot of things in Latin in my medical record*,* and used words like ‘unfortunately’ and ‘regrettably’ there.”*(OA3).

Moreover, older adults interpreted errors or weaknesses in care differently; some were more inclined to criticize and advocate for their rights following a problematic transition, while others expressed gratitude or acceptance despite shortcomings. These individual differences in what was valued most and how issues were perceived strongly influenced their sense of trust, security, and willingness to engage with the healthcare system in future transitions.

*“Maybe I would have needed someone to talk to back then*,* and someone to keep track of everything and tell me what was happening—laughs—instead of just getting papers saying*,* ‘Now you go here*,* now you go there.’ But it wasn’t something I asked for at the time. Looking back*,* I think it could have been helpful*,* but there just aren’t enough people for everything.”* (OA9).

Overall, navigating the healthcare system depended on a combination of structural factors like communication, coordination, and access to resources, along with personal factors such as prior experience, individual capacity, and social support. Several older adults shared their strategies for overcoming these challenges, including seeking information, communicating with healthcare professionals, involving relatives, and using digital services. Clear communication, coordinated planning, and personal support were crucial for feeling safe and engaged in one’s care. The system’s organization and an individual’s ability to navigate it greatly affected older adults’ experiences during care transitions.

### Theme 2, Understanding and managing one’s thoughts and emotions

This theme highlights the individual, psychological, and existential dimensions of care transitions among older adults, emphasizing their ability to adapt to aging, the health challenges that often accompany it, and the impact these changes have on their self-image. For some, the period surrounding care transitions involved near-death experiences that brought a heightened awareness of the fragile boundary between life and death, sometimes resulting in a need for therapeutic support long after discharge.

*“I guess that’s also part of why I have sought help from a counselor. Because it’s about this*,* realizing that the distance between life and death isn’t that far.”* (OA4).

*“I doubt I would be sitting here if they hadn’t found that problem.”*(OA9).

In this rural setting, older adults used various coping strategies to manage their situations and the emotional challenges of moving between different levels of care. Some relied on humor to stay motivated during rehabilitation and maintain a positive outlook for the future, while others focused on simple activities like walking. They expressed gratitude that their health could have been worse and took a day-by-day approach to managing their conditions. Additionally, a psychological aspect involved whether individuals had expected the progression of their illness before hospital admission, viewing life as a journey marked by decline, which led them to prepare proactively for unexpected health issues or the loss of function. These preparations included moving closer to healthcare facilities, selling personal belongings, or arranging legal documents such as wills or future contingency plans. The older adults believed that staying physically active was essential for recovery and maintaining independence, seeing inactivity as a faster route to decline. Simultaneously, some acknowledged the inevitability of aging and the need to downsize and adapt to new realities.

*“Life is a disease with a very poor prognosis. We will all eventually die.”* (OA6).

A common pattern among all older adults was a strong desire to remain independent and maintain control over their daily lives. Despite offers of home care services, some struggled to see how such support would be beneficial, particularly when living in remote areas where geographic distances made timely assistance challenging. This tension between autonomy and dependence was a central psychological and emotional challenge throughout the care transition experience.

*“Well*,* they did ask. I got a visit from the municipality*,* asking if I needed any help*,* like with meals or cleaning and things like that. But I said no*,* I didn’t accept any help. I want to manage on my own for as long as possible.”*(OA3).

Older adults grappled with concerns about their recovery after discharge, the fear that they might never return to their former selves, and the challenge of reconciling their current limitations with their past self-image.

*“At first*,* I thought I would become my old self again*,* but I’ve realized now that you don’t. But it could be worse.”*(OA2).

This process of adapting to a changed life after hospitalization elicited a complex range of feelings, including independence, hope, despair, uncertainty, and gratitude. Living with uncertainty and continually reassessing one’s identity were central to their experiences, and the ability to understand and manage these thoughts and emotions through coping strategies greatly affected how they viewed the quality of, and navigated, their care transitions.

### Theme 3, Feeling socially connected

This theme emphasizes the pivotal role of private relationships, social networks, and community belonging in shaping older adults’ experiences during care transitions. Social connections were vital for addressing gaps in formal healthcare while offering a sense of safety and continuity throughout the transition process. A roommate from the same locality or a familiar neighbor during hospital stays nurtured a sense of security and belonging, alleviating feelings of vulnerability in an unfamiliar environment.

*“The last time I was in the hospital*,* my neighbor from the village was there too. He passed away. Just last week*,* it was his funeral. He had cancer*,* of course. But when I was there*,* you couldn’t really tell; he was happy when I visited because we were neighbors*,* and he felt he had company. I was lucky to go home before he died.”*(OA2).

After discharge, reliance on close relatives often increased, with family members taking on significant responsibilities such as managing contact with social services or assisting with daily tasks. While this support was highly valued, it could also lead to feelings of guilt among older adults, as they were aware that their loved ones were shouldering a heavier burden. The importance of having someone to lean on became particularly evident for those who recognized that old age could bring about periods of confusion, especially in a society increasingly dependent on digital solutions, which can be challenging for individuals who, for example, forget their bank card code after hospitalization.

*“Besides things like food*,* medication*,* laundry*,* cleaning*,* and getting dressed*,* there’s also banking. When you come home feeling confused and have forgotten the code to your bank card*,* how are you supposed to buy food then? […] The importance of having a legal guardian or a close relative simply cannot be overstated.”*(OA6).

Social networks extended beyond immediate family to include friends, neighbors, and past life experiences. Early social responsibilities, such as caring for siblings during childhood, were identified as resources that facilitated resilience and recovery after discharge. Similarly, memories and imagined support from deceased parents could offer comfort and reassurance before daunting medical procedures. Neighbors played a practical role by encouraging outdoor activities and walks that aided rehabilitation and recovery. The “caring-for-neighbors” mentality, shaped by local culture, was often crucial for maintaining independence, especially when formal services were lacking or when long distances made access to care more challenging.

*“This county and its people are light-years ahead of the big cities. Here*,* there’s a real sense of community. When I’m out walking the dog in my wheelchair*,* people often ask if I need help. Sometimes I really do need a push. I get spontaneous*,* friendly offers of help.”*(OA6).

The geographical distance between home and hospital sometimes limited relatives’ ability to visit during inpatient care, requiring them to provide support from afar, often via telephone. Nevertheless, having someone to talk to regularly served as an essential safety net and source of encouragement, helping older adults recognize and celebrate progress in their rehabilitation and motivating them to accept necessary healthcare interventions. Social contacts were also crucial for logistical support, such as arranging transportation to follow-up appointments or providing accommodation in the town for multiple hospital visits.

*“Well… We stayed with some friends then*,* yes. Took the opportunity to visit while we were there. They live outside the town*,* not right in it. So we combined business with pleasure*,* as they say. Then I went for an examination. It didn’t take very long*,* maybe half an hour*,* I don’t quite remember. Afterwards*,* we returned on Thursday because I was supposed to meet the surgeon.”* (OA1).

Ultimately, these social relationships enhanced the practical aspects of daily life, access to care, and older adults’ emotional and psychological well-being. Independence was often defined not by the absence of help but by the ability to remain free from formal care services and rely on a trusted social network instead. For those living alone or with family far away, lacking this support could make everyday life and recovery after discharge significantly more challenging.

## Discussion

In this study, we explored the experiences of older adults transitioning from hospital to home in a rural context, highlighting the emotional aspects of this important process. As this study is grounded in a social constructivist paradigm, it is important to recognize that the results are co-constructed and subjective rather than an objective truth. The findings are presented as participants’ subjective experiences of transitional care, rather than definitive facts. This highlights the importance of reflexivity and transparency throughout the research process to enhance the credibility of the findings. While the concept of transferability is a concern in some qualitative methodologies, within Reflexive Thematic Analysis, it is the contextual richness and reflexivity that support the applicability of findings rather than generalized transferability [52]. While Meleis’ Transition Theory [1] contributed important concepts such as transitional care and participation, the study’s theoretical framework was also informed by other relevant literature and empirical research. These combined perspectives served as a foundation for the research questions and analytic orientation, thereby facilitating reflexivity and depth in understanding this complex phenomenon. While alternative theoretical perspectives might illuminate other facets of transitional care, the adopted perspective provides coherence and depth in understanding this complex, multifaceted phenomenon.

The results show that transitional care is a complex phenomenon, and the older adults’ experiences with transitional care are influenced by organizational, psychological, and social factors. These experiences are highly personal, largely influenced by individual resources and situations. Additionally, the findings emphasize the urgent need for registered nurses and other healthcare professionals to perform thorough assessments that consider each older adult’s specific abilities and needs during these transitions, considering the unique organizational, psychological, and social dimensions. Therefore, building a strong relationship with older adults, even during short hospital stays, is both beneficial and essential. This study demonstrates that care transition experiences vary widely and are greatly affected by various elements, including personal history, psychological resilience, and the level of social support available.

Some older adults reported feelings of acceptance and gratitude, while others experienced frustration, uncertainty, and mistrust toward the healthcare system. This diversity supports the conclusions of Andreasen et al. (2015), highlighting older adults’ increased vulnerability to feelings of insecurity during transitions. Additionally, these findings strengthen Meleis’ Transition Theory, which argues that transitions are inherently uncertain and require a tailored approach to support, promoting adaptation and well-being (Meleis, 2010; Schumacher & Meleis, 1994). Notably, these transitions are multi-layered, involving simultaneous processes like changes in the physical environment and shifts in self-perception. This emphasizes the importance of addressing both personal identity (“the ‘I’” of the older adult) and contextual factors (such as geographical location) in care planning.

In light of these compelling insights, healthcare professionals must reject the idea of a one-size-fits-all strategy and fully commit to understanding each individual’s resources, needs, and preferences, meticulously tailoring their communication, planning, and support accordingly. This approach aligns with the core principles of person-centered care and is crucial for enhancing the quality of care transitions [13, 36]. The core principles of person-centered care involve working in partnership with the older adult, learning from each other’s experiences, and sharing information, deliberation, and decision-making, based on the older adult’s preferences, beliefs, and values [53]. The person-centered approach is not only fundamental for improving care transitions but is also a prerequisite for achieving the goals of the ongoing Swedish health care reform, ‘good quality, local health care’. The reform seeks to realize high-quality, locally coordinated, participatory care, strengthening equity, continuity, and collaboration within the decentralized Swedish health care system [54, 55]. Most people expect to participate actively in decisions affecting them, including healthcare decisions [56]. However, our study shows that it is important to determine how active and in what way the individual wants to be and can be. By deepening the understanding of the individual undergoing transition, the quality of care can be substantially improved, ensuring it meets the unique and dynamic needs of older adults in rural settings.

Our findings reveal that a robust social network is a protective factor, enhancing practical navigation within a fragmented healthcare system and providing essential practical and emotional support. Nonetheless, not all older adults have access to strong social support, particularly in rural areas where geographic isolation and resource constraints are prevalent [22, 24]. This disparity raises critical concerns about equity in care transitions, as those lacking informal support may encounter poorer outcomes. Consequently, healthcare professionals must proactively identify individuals at risk of social isolation and deliver additional support, echoing the recommendations of Ingvarsson et al. [9].

The rural context introduces unique structural barriers, including long geographical distances to healthcare facilities, limited transportation options, and fragmented responsibilities among care providers [21, 22]. Previous research indicates that these factors can exacerbate feelings of confusion and abandonment [7, 8]. Our results indicate that older adults in rural contexts face transportation challenges and difficulties navigating the healthcare system, reinforcing findings in the international literature on rural care transitions [28, 30, 32]. To effectively address these challenges, we advocate for improved coordination and communication among care providers, flexible transportation solutions, and tailored follow-up procedures specific to the rural context. The importance of clear, accessible information and continuity of care cannot be overstated [9, 24].

Our results strongly support existing theories highlighting communication as a pivotal factor. Older adults who felt well-informed and involved in planning their transitions reported greater confidence and lower anxiety levels. In contrast, those who experienced poor communication felt marginalized and insecure, consistent with previous findings indicating that older adults in Sweden frequently feel excluded from healthcare- and discharge planning, whether in urban or rural settings [9, 14].

The transition from hospital to home transcends a mere physical relocation; it encapsulates a psychological process involving shifts in self-image and identity. The older adults in our study articulated their struggles and triumphs in adapting to new limitations and realities, often necessitating emotional support and clear information. This aligns with Meleis’ [1] assertion that transitions represent vulnerable periods wherein the risk of adverse outcomes is heightened if not adequately supported. Furthermore, our study elucidates that being an older adult navigating transitions in rural environments entails facing challenges such as transportation difficulties and issues with healthcare navigation, while also benefiting from socio-cultural support through networks of contacts.

Our results resonate with previous Swedish and Scandinavian qualitative research on older adults’ experiences of care transitions [7, 9, 10, 57, 58], confirming the impact of healthcare system organization, the importance of social support, and the profound life changes experienced by older adults following hospitalizations. This study adds to the extant literature by in-depth exploring the rural Swedish context. It elucidates how healthcare organizations and resource distribution distinctly affect individuals living long distances from health services and how social networks both shape and are shaped by geographic remoteness. Most notably, the results demonstrate that organizational, psychological, and social aspects of care transitions are fundamentally intertwined, dynamically influencing one another throughout the transition process. Rather than viewing these factors in isolation, this study highlights their complex interaction, which is particularly salient in rural settings. This interdependence is a novel contribution to the literature, extending the understanding of care transitions as deeply contextual and multidimensional phenomena.

### Implications for practice

This study’s findings suggest that healthcare professionals must thoroughly assess each older adult’s specific circumstances, including their psychological resources and social networks. This is in line with person-centered care and the goals of the Swedish “good quality, local care reform,” and such evaluations are crucial for identifying and supporting individuals at risk of social isolation or lacking informal support systems. Healthcare professionals can implement brief social-network and transport checklists at hospital discharge to systematically assess individuals’ supportive networks and practical barriers.

Moreover, the study highlights that care transitions encompass more than transferring older adults from hospital to home. Thus, it is imperative for healthcare personnel to acknowledge the psychological dimensions associated with these transitions and to provide necessary emotional support. Discharge teams should use scripts for preference-sensitive information delivery, tailoring both format and content to each older adult’s expressed worries, coping needs, and information preferences.

Additionally, practitioners should deliver personalized information and actively engage older adults in the care planning processes. This engagement is essential for ensuring effective coordination and continuity of care, particularly in rural contexts where healthcare resources may be limited. It is recommended to set triggers for post-discharge follow-up among older adults living in very remote locations or those flagged at discharge as lacking adequate informal or formal support.

While challenges such as transportation barriers are more acute in rural areas, the fundamental findings related to social support and psychological adaptation likely have broad relevance. The capacity to benefit from robust support networks and effective coping mechanisms during care transitions may not be exclusive to older adults residing in rural regions, indicating that interventions focusing on these areas can improve outcomes across various settings.

### Strengths and limitations

While the strength of providing an in-depth understanding of an under-researched context is notable, the study is limited by its small sample size. The findings indicate that experiences vary significantly from person to person based on individual factors, and the small sample may mask even greater variations in how care transitions are perceived among older adults in rural areas. Nevertheless, the results highlight distinctive aspects such as the necessity of reliable transportation to and from healthcare services when facing challenges related to long geographical distances, which are not typically associated with urban environments. Therefore, the study contributes valuable insights into how care transitions are experienced by older adults in a rural context, an area where further research is needed. These points emphasize the need for more research in this field to enhance knowledge and enable optimal individual adaptation of health and social care services.

While multiple recruitment pathways were utilized, including direct outreach via healthcare staff in clinics and hospitals, as well as advertisements in community centers and on social media, it remains possible that the most frail older adults, lacking strong social networks or digital access, may have been underrepresented in this study. Recruiting through health professionals enabled participation even among those with limited social contacts; however, barriers such as mobility limitations and digital exclusion might have influenced who was reached, potentially shaping the relative prominence of system navigation experiences compared to overt structural barriers.

The repeated interviews conducted with some participants enhanced the information power by allowing for a deeper understanding of the older adults’ experiences. Without the opportunity to conduct follow-up interviews sometime after discharge, a larger sample size may have been necessary to achieve sufficient information power to explain the studied phenomenon comprehensively. This approach aligns with the principles advocated by Braun and Clarke [40] and Malterud, Siersma [45], emphasizing the depth and richness of data over mere sample size.

The older adults were offered the choice of interview time and mode (face-to-face or phone), which limited the observation of non-verbal cues such as facial expressions and body language in phone interviews. However, when comparing interviews during analysis, no difference in the richness or depth of content was identified across the interview approaches. This flexibility can be considered a strength, as Elwood and Martin [59] suggest that allowing participants to choose interview conditions can foster comfort, openness, and empowerment in the research interaction.

## Conclusions

In conclusion, this study advances the field by showing that care transitions for older adults in rural Sweden are not merely shaped by discrete structural, psychological, or social influences, but by the intricate interplay of these factors, an interaction that is critical for informing tailored, context-sensitive support and intervention strategies in similar settings. To ensure safe and supportive transitions, healthcare professionals need to go beyond standard routines and focus on understanding and addressing each individual’s unique assets and needs in these dimensions. Otherwise, older adults might develop mistrust toward the healthcare system and, in worst cases, be reluctant to seek care when necessary.

## Supplementary Information

Supplementary Material 1

## Data Availability

The datasets generated and analysed during the current study are not publicly available due to privacy and ethical restrictions, but are available from the corresponding author on reasonable request.

## Footnotes

1 Defined as persons aged 65 years and older [3].

2 Idun Byberg

3 OA referring to Older Adult.
