# Correction: Older adults’ experiences of hospital-to-home transitions in rural Sweden: a qualitative study

**DOI:** 10.1186/s12877-026-07626-0

**Published:** 2026-05-28

**Authors:** Idun Byberg, Ulla Näppä, Marie Häggström

**Affiliations:** 1 Department of Health Sciences, Mid Sweden University, Östersund, S-831 25 Sweden; 2 Department of Health Sciences, Mid Sweden University, Sundsvall, S-851 70 Sweden

**Correction: BMC Geriatr 26**,** 18 (2026)**

10.1186/s12877-025-06780-1

Following publication of the original article [1], the author requested a correction in the author name and email address of Idun Byberg.

Previous author name: Idun Winqvist

Old initials: I.W.

Previous email address: idun.winqvist@miun.se

New author name: Idun Byberg

New initials: I.B.

New email address: idun.byberg@miun.se

The author group has been updated above and the original article [1] has been corrected.
