# Supplementary material for: Older adults’ experiences of hospital-to-home transitions in rural Sweden: a qualitative study
Source: BMC Geriatr. 2025 Dec 2;26:18. doi: 10.1186/s12877-025-06780-1 (PMC12777439; doi:10.1186/s12877-025-06780-1)
Supplement: Supplementary file 1 — Supplementary Material 1 [file 12877_2025_6780_MOESM1_ESM.docx]

| Interview text | Semantic coding | Latent interpretation |
| --- | --- | --- |
| He has been the boss at the primary care center all these years. He said, "I'll put it like this. I don't have any woman as old as you, who doesn't have half a handbag of medicine at your age". Because that's how it is. No, I said, maybe I need something to calm me down. But no one has noticed. No, I haven't had any medicine. I haven't been sick. I haven't been sick at all. ***(OA7)*** | There is no woman my age who doesn't have half her handbag filled with medicines, but I haven't been sick and haven't needed any. | In comparison to others my age, I’ve been healthy. |
| That's the disadvantage when you have knee surgery because they're made of metal. So, you can never kneel. You can't have the metal against your kneecap. Then you could crack your kneecap. So now you must remember to wear knee pads. So now I'll just wear knee pads. In the garden. And. Knee pads. I have good, padded ones that I bought when I danced a lot. They're really good. ***(OA8)*** | A disadvantage of having had knee surgery is that I can never kneel, I must always wear knee pads in the garden. | Lifelong adaptation and altered daily life because of the medical intervention. |
| ***OA2’s spouse***: Do you need a straw [for your coffee]?  ***Interviewer***: No thanks, I'm fine *laughs*.  ***OA2:*** But it's great when you're shaky.  ***Interviewer***: Yes, I understand that.  ***OA2***: Really!  ***Interviewer***: So, you’ve bought a small supply now?  ***OA2***: Yes.  ***Interviewer***: Because of your shakiness?  ***OA2***: Yes, *laughs*, *spouse* says it was such a big package. But now I see we might...  ***OA2’s spouse***: One hundred were in it. | The shakiness entails that I must drink coffee with a straw. | Adaptation in daily life following the hospital stay. |
| **Reflexive memos belonging to Theme 2, Understanding and managing one’s thoughts and emotions** | | |
| A prominent aspect spanning multiple (OA1, OA3, OA4, OA5, OA6, OA8) is the anxiety and uncertainty about the future. While some (OA5, OA8) adopt a coping strategy of acceptance and positive reframing, others (OA3, OA4) experience significant distress, reflected in OA4’s need to seek counseling post-discharge and OA3’s anxiety following the loss of a close friend. The notion of ‘stubbornness’ or ‘persistent self-advocacy’ emerges as a shared yet nuanced coping mechanism by OA3, OA4, OA5, and OA8, highlighting varying degrees of empowerment or perceived burdensomeness in healthcare interactions. This dynamic interplay raises questions about how trust in healthcare and being heard impacts emotional resilience. | | |
| Participants reveal contrasting approaches to preparing for age and health decline. OA2 and their relatives proactively relocated closer to health services and organized futures planning, including legal safeguards, whereas OA7’s experience illustrates the distressing forced abandonment of cherished home environments due to illness-related adaptations. OA6 and OA9 also emphasize the importance of future directives and planning to mitigate challenges posed by possible cognitive decline. Variability also surfaces in acceptance of external help, with OA7 engaging support willingly, while OA3 resists despite acknowledged needs. This spectrum of reactions underscores the complexity of navigating autonomy, dependence, and existential contemplation. | | |

### Appendix 1. Examples from the analysis and of reflexive memos

Table A1. Examples of coding of excerpts later sorted into Theme 2, Understanding and managing one’s thoughts and emotions


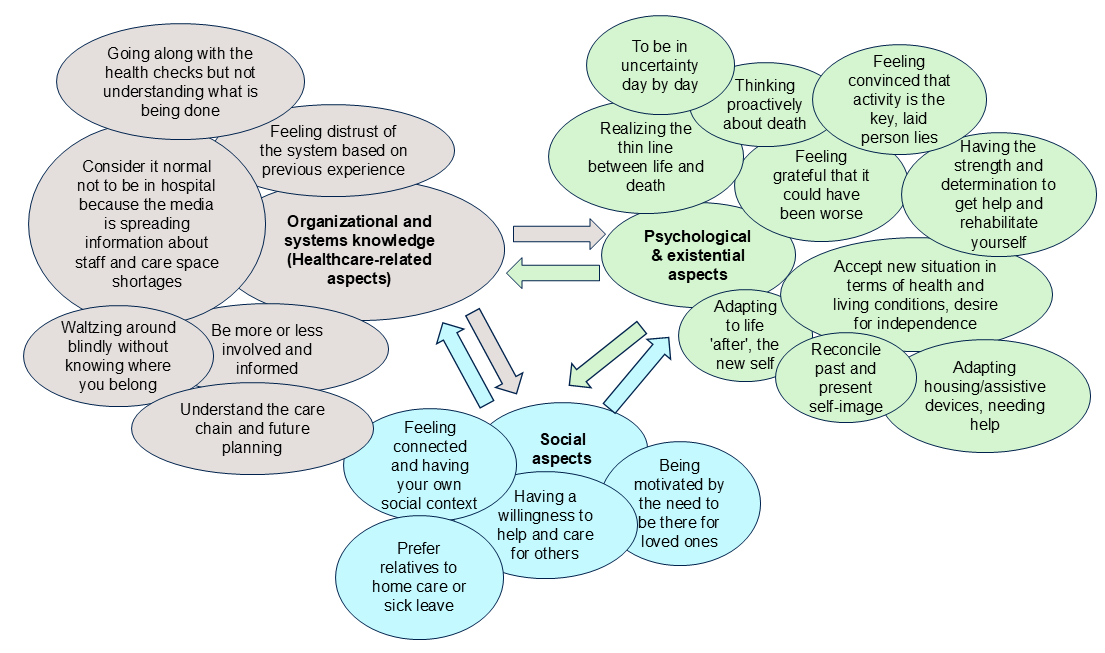
Figure A1. An excerpt from the initial code sorting using PowerPoint

## **Example of a reflexive memo regarding interviewing**

As I (I.W.) possess knowledge about how older adults can get help with unresolved, care-related problems, it has felt ethically wrong not to assist with advice on how they can address these issues. For example, OA5 did not know whether a prescription had been forgotten or if she was assessed as not needing the treatment they discussed at the follow-up in primary care. In this situation, I slipped into my role as a district nurse and advised her to contact primary care to find out if a prescription had been missed. Suddenly, I found myself not only in the interview situation as a researcher, but also as a well-meaning district nurse with my experience of how easily a prescription can be missed. In terms of power dynamics, this might skew the interview situation because I depend on the older adults being open about their experiences, while simultaneously reminding them that I am trained and knowledgeable about some of what they share. However, this has been discussed within the research team, and we have concluded that it is the most ethically correct action in the situation to share my knowledge when older adults are already sharing their experiences, while finding themselves in an undesirable position.

OA4 saw the interview as somewhat therapeutic; when she had not been able to get in touch with healthcare for answers to her questions, she was instead grateful to be able to talk to me the same day, as I am at least healthcare personnel by background and listen to her concerns, even if I cannot provide answers. In this way, the interview situation differed markedly compared to when I interviewed retired healthcare professionals. For example, the older adult who seemed to identify more as a retired healthcare professional, now being an older adult, rather than as an older adult also being a retired healthcare professional, the conversation took a more collegial tone regarding the use of terms. Thus, my role as an interviewer has had to shift depending on my experiences, the older adults' experiences, and what they know about my education and research. It has felt somewhat difficult to be entirely in the researcher role, as, in the conversation, my district nurse mindset also kicks in.
